# An Experimental Examination of the Interaction between Mood Induction Task and Personality Psychopathology on State Emotion Dysregulation

**DOI:** 10.3390/bs5010070

**Published:** 2015-03-09

**Authors:** Lauren M. Borges, Amy E. Naugle

**Affiliations:** Department of Clinical Psychology, Western Michigan University, 3700 Wood Hall, Kalamazoo, MI 49008, USA; E-Mail: amy.naugle@wmich.edu

**Keywords:** emotion dysregulation, personality disorder, behavioral analogue, mood induction

## Abstract

While emotion dysregulation has been investigated as a key variable in the development and persistence of personality psychopathology, few studies have explored state emotion dysregulation among individuals with personality disorders (PDs). The current study addresses this void in the literature through a laboratory investigation of state emotion dysregulation among participants with and without PDs. To facilitate this goal, participants were matched to pairs based on similar personality features and were randomized to one of two behavioral analogues; either the Paced Auditory Serial Addition Task-Computerized (PASAT-C) or an interpersonally based mood induction. As hypothesized, PD participants in the PASAT-C reported significantly more difficulty with impulsivity and emotion regulation strategies. Contrary to expectations, the PD group in the interpersonal task demonstrated significantly less difficulty with non-acceptance of emotion and emotional clarity and significantly greater positive affect compared to non-PD participants. Implications for these findings and directions for future research are discussed.

## 1. Introduction

Personality disorders (PDs) can be conceptualized as disorders of emotion dysregulation. In fact, each personality disorder in the DSM-5 includes at least one symptom related to emotional dysfunction. Experimental studies have established emotion dysregulation as a mechanism underlying Borderline Personality Disorder (BPD), Antisocial Personality Disorder (ASPD), Histrionic Personality Disorder (HPD), Narcissistic Personality Disorder (NPD), Obsessive Compulsive Personality Disorder (OCPD), and Avoidant Personality Disorder (AVPD) [1,2,3,4,5,6,7,8,9,10]. In the DSM-5, emotion dysregulation is even more clearly articulated as a core feature of personality psychopathology in the criteria for “general personality disorder” [11]. To meet criteria for a “general personality disorder”, an individual must exhibit an “enduring pattern of inner experience and behavior that deviates markedly from the expectation of the individual’s culture”, a pattern which is applied inflexibly and causes clinically significant distress or impairment in functioning. Among these inner experiences and behaviors are difficulties associated with “cognition (*i.e.*, ways of perceiving and interpreting self, other people and events)”, “affectivity (*i.e.*, the range, intensity, lability, and appropriateness of emotional response)”, “interpersonal functioning”, and/or “impulse control” [11].

Theoretical models of emotion dysregulation account for the symptoms of “general personality disorder” and highlight the role of emotion dysregulation in the development and maintenance of PDs in general. The most well established model of emotion dysregulation in the personality psychopathology literature is Gratz and Roemer’s [12] theory of emotion dysregulation. Within this line of research, emotion dysregulation has been established as a mediating variable central to the development and maintenance of PDs [1,2,3,4,13,14]. Gratz and Roemer [12] identify non-acceptance of emotions, lack of emotional awareness, difficulty engaging in goal directed behavior when distressed, lack of access to situationally appropriate emotion regulation strategies, impulsivity, and lack of emotional clarity as dimensions of emotion dysregulation. While these categories are all indicative of persistent problems with “affectivity” and the category “impulse control difficulties” is explicitly addressed through one facet of Gratz and Roemer’s definition of emotion dysregulation, difficulties with “cognition” and “interpersonal functioning” are also accounted for within this model. For example, if an individual is experiencing emotion dysregulation to a great degree, that person may demonstrate difficulties engaging in goal directed behavior (e.g., difficulty setting a boundary in a relationship) indicative of interpersonal dysfunction due to emotion dysregulation. Difficulties associated with “cognition” may present in PDs through the experience of distressing thoughts indicative of non-acceptance of emotions (e.g., thoughts like, “I am weak for showing emotion”).

While this research emphasizes the role of trait emotion dysregulation in personality disorders, these studies do not account for state emotion dysregulation as a multidimensional construct. The use of specific emotion regulation strategies across different situational contexts is also not considered in this line of research. Measuring state based emotion dysregulation may be useful in understanding the interaction between environmental triggers and emotional responding. Few studies have explored state emotion dysregulation among individuals meeting criteria for PDs. The present study addresses this gap in the extant literature through a laboratory investigation of state emotion dysregulation among individuals with and without PDs.

In order to investigate the role of state emotion dysregulation across PDs, laboratory based mood induction procedures are often implemented to facilitate the measurement of emotional responding. The Paced Auditory Serial Addition Task-Computerized version (PASAT-C) is one commonly employed laboratory task with mood-inducing properties [15]. The PASAT-C was originally developed as a neuropsychological assessment of information processing and capacity for patients with histories of head trauma. This task has been subsequently revised and applied as a measure of distress tolerance. In order to assess willingness to tolerate distress, the PASAT-C must evoke emotional distress. Research has demonstrated that increased anxiety, anger, frustration, irritability, and “general distress”, often measured as anger and irritability, have been induced from the PASAT-C across participants [15].

During the PASAT-C, participants are instructed to add math problems in a computer task at an increasing rate in the presence of an explosive sound. Towards the end of the task, the option to terminate the task is presented. Participants meeting criteria for personality psychopathology (*i.e.*, BPD, ASPD, and AVPD) demonstrate heightened unwillingness to tolerate distress when engaged in the PASAT-C by terminating the task earlier than healthy controls [12,15,16,17,18]. Task termination time measures willingness to pursue goal directed behavior when distressed, one component of Gratz and Roemer’s [12] definition of emotion regulation. A complete assessment of emotion regulation, however, is not afforded by the PASAT-C. In order to evaluate more components of state emotion regulation, self-report measures can be administered immediately before and after this mood induction task.

One problem with relying on the PASAT-C to research emotion regulation in PDs is that its generalizability to the specific struggles of individuals with personality psychopathology may be limited [16]. Interpersonally based behavioral analogues may be more relevant to PDs, as interpersonal dysregulation has been identified as a core feature of personality psychopathology [19]. Therefore, understanding an individual’s response to an interpersonally based stressor could be more relevant to improving that individual’s quality of life. To investigate how individuals with PDs regulate emotions in interpersonal contexts, laboratory based social rejection scenarios are often implemented. In one such study, participants were asked to audio record a 5-minute personally applicable social rejection scenario and were instructed to listen to that recording. Following this social rejection task, individuals with more BPD symptoms demonstrated heightened negative affect and more difficulty applying appropriate social problem solving skills [20]. Another study found that personally relevant social rejection imagery was more evocative in inducing distress and dissociation across participants (BPD and controls alike) than a standardized rejection video [21].

While these kinds of mood inductions are effective in eliciting emotion dysregulation in an interpersonal context, reliance on past rejection scenarios may be problematic. Studying emotion regulation in the context of previously experienced social rejection may be confounded by prior rehearsal of emotion regulation strategies. To minimize the effects of rehearsal and ensure the personal relevance of the mood induction, novel and *in vivo* social rejection tasks can be implemented [22,23]. One example of a novel and *in vivo* social rejection mood induction is Bushman and Baumeister’s [24] abortion essay writing task. In this task, participants are asked to write an essay on their abortion stance and are provided a negative evaluation of their essay by a “partner” who is really a research assistant acting as a confederate. This task has been associated with increased anger and hostility among participants [24].

### The Current Study

The present study was developed to address two primary goals. The first goal was to directly compare two contextually different mood induction tasks to determine if the type of behavioral analogue evoked different features of state emotion dysregulation among individuals with and without a PD. The two mood induction conditions employed were the PASAT-C and an interpersonal mood induction, Bushman and Baumeister’s [24] essay writing task. Both tasks were implemented to induce upset feelings and emotion dysregulation in participants. Preexisting studies have not directly compared an interpersonal mood induction to the PASAT-C to understand its effects on state emotion dysregulation among individuals meeting criteria for personality psychopathology. The second goal of this study was to assess state emotion dysregulation among individuals with and without PDs using self-report measures. As described earlier, several studies have assessed state difficulties engaging in goal directed behavior when distressed among individuals with PDs, but to our knowledge, no study has measured state emotion dysregulation in PDs as a multidimensional construct.

Based on the goals of the present study, we formulated the following hypotheses:

1. The PASAT-C and the essay-writing task would evoke different emotion regulation strategies.
2. Individuals with personality disorders would respond with more emotion dysregulation to each mood induction when compared to individuals without PDs.
3. Based on these hypotheses, we believed the following would result in the interaction between mood induction condition and personality disorder status:
  - Individuals meeting criteria for a PD in the PASAT-C would report more difficulties with impulsivity, goal directed behavior, and lack of access to appropriate emotion regulation strategies (as measured by the S-DERS) following the task than all other participants. Individuals meeting criteria for a PD in the PASAT-C would not respond with heightened dysregulation in the other domains of emotion dysregulation when compared to the essay-writing condition.
  - Individuals meeting criteria for a PD in the essay-writing task would respond with more difficulties with non-acceptance of emotions (S-DERS), emotional clarity (S-DERS), emotional awareness (S-DERS), and suppression (S-ERQ) than all other participants. Given the nature of the essay-writing task, it was not expected that this procedure would evoke difficulties with goal directed behavior, emotion regulation strategies, or impulsivity.

In previous studies employing the PASAT-C and the trait version of the DERS, individuals meeting criteria for PDs demonstrated heightened unwillingness to engage in goal directed behavior when distressed [16,17,18]. We hypothesized that the effects of the PASAT-C on emotion dysregulation would be greatest in the domains of impulsivity, goal directed behavior, and strategies because of the immediately disruptive nature of the task (e.g., briefly flashing numbers and explosive sounds). Because individuals meeting criteria for PDs often demonstrate skill deficits in interpersonal contexts, we hypothesized that it would be more difficult for these participants to notice, accept, and understand emotions in response to social rejection. Based on this, we hypothesized that participants with a PD in the essay-writing condition would respond to the task with more state difficulty in the domains of non-acceptance of emotions, emotional clarity, awareness of emotions, and emotional suppression compared to all other participants. We did not expect that participants in the essay-writing task (PD or no PD) would respond with elevated difficulties in impulsivity, goal directed behavior, or difficulties accessing emotion regulation strategies as the task was not immediately disruptive in nature.

## 2. Method

### 2.1. Participants

Participants were undergraduate students recruited from courses at a large Midwestern university. The sample includes 174 participants who completed both sessions of the study. Of these participants, 70.9% were women and ranged in age from 18–41 with a mean age of 20.34 (*SD* = 3.29). With regard to race/ethnicity, 5.7% (*n* = 10) of the sample identified as Asian, 12% (*n* = 21) African American, 3.4% (*n* = 6) Hispanic, 74.3% (*n* = 130) Caucasian, and 4.0% (*n* = 7) biracial (see Table 1). Participants were matched according to personality dimensions and personality disorder status on the SNAP-2 and then assigned to either the PASAT-C condition (*n* = 85) or the essay writing condition (*n* = 90). Of the participants included in the analyses, 112 individuals did not meet criteria for a PD. Sixty-three participants met DSM-V criteria for a PD (*n* = 40 women). While this reflects a high rate of PD diagnosis, rigorous epidemiological studies have found that 18% of college students meet criteria for a PD through a structured diagnostic interview [25]. The use of a self-report PD measure (although validity measures were included) and oversampling of participants from undergraduate psychology coursework may have contributed to an inflation of PDs in the present sample.

**Table 1:** Demographic information with ANOVAs and chi-square analyses.

*n* = 174	PASAT-C *n* = 85	Essay *n* = 89	PD *n* = 63	No PD *n* = 111	Significance (Condition, PD)
Mean age standard deviation	20.56 (3.53)	20.12 (3.04)	20.42 (3.79)	20.54 (3.53)	*F*_condition_ (1, 169) = 0.78, *p* = 0.37 *F*_PD_ (1, 169) = 0.62, *p* = 0.43
Sex (% female)	76.30% (0.45)	68.90% (0.46)	62.30% (0.48)	62.30% (0.48)	χ^2^_condition_ (1) = 0.23, *p* = 0.74 χ^2^_PD_ (1) = 2.14, *p* = 0.16
Race (% Caucasian)	72.10% (1.32)	75.60% (1.37)	77.30% (1.29)	73.30% (1.38)	χ^2^_condition_ (4) = 2.59, *p* = 0.63 χ^2^_PD_ (4) = 1.38, *p* = 0.85
Education (% freshman)	41.90% (1.23)	45.60% (1.31)	48.10% (1.22)	38.90% (1.26)	χ^2^_condition_ (4) = 3.91, *p* = 0.42 χ^2^_PD_ (4) = 2.95, *p* = 0.57
Income (% ≥ $15,000)	88.40% (0.54)	88.90% (0.54)	79.20% (0.74)	91.60% (1.15)	χ^2^_condition_ (3) = 1.50, *p* = 0.68 χ^2^_PD_ (3) = 2.27, *p* = 0.52
Residence (% dorms)	45.30% (1.33)	47.80% (1.29)	51.90% (1.34)	42.70% (2.80)	χ^2^_condition_ (5) = 6.32, *p* = 0.28 χ^2^_PD_ (5) = 5.38, *p* = 0.37
PD Status (% no PD)	60% (0.49)	67% (0.47)	-	-	χ^2^_condition X PD_ (1) = 1.15, *p* = 0.35

Note: no significant differences between groups were found.

### 2.2. Measures

The present investigation is part of a larger measure development study. As such, the measures included in this article are specific to questions regarding the effects of mood induction condition on state emotion dysregulation in personality psychopathology.

#### 2.2.1. Assessment of Personality Psychopathology

The Schedule for Nonadaptive and Adaptive Personality-2 (SNAP-2) is a 390-item, true or false, self-report measure of normal and abnormal personality features [26]. The SNAP-2 assesses DSM-V personality disorders in addition to 12-lower order personality dimensions, three higher order temperament scales (negative temperament, positive temperament, and disinhibition), and includes seven validity scales. The present study employed a categorical diagnostic scoring approach to the assessment of PDs using the DSM-V PD criteria within the SNAP-2. Diagnosis of PDs was determined based on meeting criteria for one or more PD diagnoses. To obtain a PD diagnosis, test items assess whether or not an individual endorses a specific criterion within a PD diagnosis and then whether that individual endorses enough criteria to warrant a PD diagnosis. The SNAP-2 includes 24 items assessing Paranoid PD (four of seven criteria required for a diagnosis), 21 items for Schizoid PD (four of seven criteria required), 25 items for Schizotypal PD (five of nine required), 34 items for Antisocial PD (three of seven required), 33 items for Borderline PD (five of nine required), 23 items for Histrionic PD (five of eight required), 25 items for Narcissistic PD (five of nine required), 19 items for Avoidant PD (four of seven required), 20 items for Dependent PD (five of eight required), and 25 items for Obsessive Compulsive PD (five of eight required). High rates of internal consistency are demonstrated within the personality disorder scales with alpha coefficients ranging from 0.69 (obsessive-compulsive) to 0.88 (avoidant). Stable test retest reliability has been established at one-week follow-up (0.81–0.93) and moderate test retest reliability is found at four months (0.76–0.89) [26]. Four trait scales of neuroticism, conscientiousness, introversion, and antagonism have been identified within the SNAP-2 as the SNAP “Big Four”, which correlate with four of the “Big Five” factors demonstrated in the NEO Five Factor Inventory (NEO-FFI) and the Personality Pathology-5 (PSY-5) scales of the Minnesota Multiphasic Personality Inventory-2 (MMPI-2) [27]. In the current study, internal consistency ranged from 0.65 (obsessive-compulsive) to 0.83 (avoidant and borderline).

#### 2.2.2. State Affect Measure

The Positive and Negative Affect Schedule (PANAS) is a 20-item self-report measure used to measure positive affect (PA) and negative affect (NA) [28]. Participants provide a mood rating for each item ranging from “1”, which indicates experiencing a mood state “very slightly or not at all” to “5” which is “extremely” relevant to the participant’s mood state. Research suggests a 3-factor version of the PANAS may account for more mood variability in college student samples than the typical two-factor (PA and NA) approach. In the 3-factor model, the “NA” scale is separated into two lower order factors of “upset” and “afraid” [29,30]. The “upset” scale is comprised of the items “irritable”, “ashamed”, “guilty”, “hostile”, “upset”, and “distressed” and is associated with anger and dejection. Related to anxiety, the “afraid” scale includes the items “scared”, “nervous”, “jittery”, and “afraid”. Good internal consistency (ranging from 0.72–0.86) has been found for the three-factor version of the PANAS. Convergent validity has also been demonstrated, as the three-factor PANAS has been compared to the Beck Depression Inventory (BDI) and the Pleasure Arousal and Dominance (PAD) emotional scales [29,30]. The three-factor scoring procedure was applied to the present study. In the current study, good to excellent internal consistency was demonstrated within the upset (α = 0.74), afraid (α = 0.86), and positive affect (α = 0.90) subscales.

#### 2.2.3. State Emotion Regulation Measures

The State*-*Emotion Regulation Questionnaire (S-ERQ) [31] is a state version of Gross and John’s [32] trait based ERQ. The S-ERQ is an eight-item measure designed to measure in the moment emotion regulation strategies. Consistent with Gross’s theory of emotion regulation, state-based suppression (e.g., “I controlled my emotions by not expressing them”) and reappraisal (e.g., “I controlled my emotions by changing the way I thought about the situation I’m in”) are the regulatory strategies assessed with this measure. Participants are instructed to rate agreement with items on the ERQ on a 1 (strongly disagree) to 7, (strongly agree) scale. Kashdan *et al.* [31] found acceptable convergent validity in examining the relationship between the trait ER and state ERQ. Excellent reliability has been demonstrated by the S-ERQ in both the suppression (0.97) and reappraisal (0.97) subscales in past research [29]. In the current study, good internal consistency was found for the suppression (0.84) and reappraisal (0.85) subscales of the S-ERQ.

The State Difficulties in Emotion Regulation Scale (S-DERS) [33] is a state version of Gratz and Roemer’s [12] trait based DERS. The S-DERS is a 30-item questionnaire designed to assess state-based emotion regulation difficulties consistent with Gratz and Roemer’s [6] theory of emotion regulation. The version of this measure used in the present study was comprised of five subscales including non-acceptance of emotions (e.g., “I feel guilty for feeling this way”), difficulty engaging in goal directed behavior (e.g., “I am having difficulty focusing on anything other than my emotions”), impulse control difficulties (e.g., “I feel out of control”), lack of awareness of emotions (e.g., “I am paying attention to how I feel”), difficulty accessing emotion regulation strategies (e.g., “I am feeling very bad about myself”), and lack of emotional clarity (e.g., “I have no idea how I am feeling”). Since its inclusion in the current study, the S-DERS was revised to a four-factor model. A new subscale, “modulate”, was created from the impulse, goals, and strategies subscales. Lavender *et al.*, (under review) found good internal consistency for the total scale (α = 0.86), adequate to excellent internal consistency for the non-acceptance (α = 0.92), modulate (α = 0.85), and awareness subscales (α = 0.79), and satisfactory internal consistency for the clarity subscale (α = 0.65). Construct validity and predictive validity have also been established for the S-DERS when compared to the trait version of the DERS. Additionally, the S-DERS demonstrated discriminant validity when compared to the Affect Intensity Measure [34] and the Five Facet Mindfulness Questionnaire [35]. In the current study, internal consistency rates were generally acceptable for the non-acceptance (α = 0.94), impulse (α = 0.62), goals (α = 0.62), awareness (α = 0.62), strategies (α = 0.81), and clarity (α = 0.67) subscales of the S-DERS.

### 2.3. Mood Induction Conditions

The Paced Auditory Serial Addition Task (PASAT-C) is a measure of distress tolerance that has been demonstrated to evoke negative affect in individuals [15,16]. The PASAT-C is a serial addition task where numbers are sequentially flashed on a computer monitor. Participants are instructed to add the most recently presented number to the previously displayed number and select the correct answer. After an answer is provided, that response must be disregarded, as participants are instructed to add the next number flashed to the most recently presented number. Participants are informed that they will receive 1 point for every correct answer selected, which corresponds with a bank of their total points at the top of the PASAT-C screen. If an incorrect sum is selected or an answer is not chosen before the presentation of the next number, an “explosion” sound is emitted. The latency between levels becomes increasingly abbreviated in the PASAT-C, making the task more challenging as participants advance. In this version of the PASAT-C, level 1 had a 3-second latency between flashed numbers (level 1 duration was 3 min), level 2, a 2-second latency (level 2 duration was 5 min), and level 3, a 1-second latency (level 3 duration was up to 7 min). During the third level of the task, participants had the option to terminate the task at any time.

Bushman and Baumeister’s [24] essay writing task is a mood induction procedure that often evokes anger and hostility among participants [36]. This task induces mood through social rejection. Participants are asked to write a one-paragraph essay on their abortion stance (either pro-choice or pro-life), which will be evaluated by a “partner” for the purposes of understanding “first impressions”. In reality, the assigned partner is a confederate research assistant who evaluates the participant’s essay and provides standardized negative feedback. Included in this feedback is the comment “this is the worst essay I have ever read” and an evaluation in the domains of organization, originality, writing style, persuasiveness of arguments, and overall quality. The rating form’s scale ranges from −10 (very bad) to +10 (very good) and the participant attains scores ranging from −10 to −8 in all domains evaluated. Participants are given a time limit of 5 min to write this essay. While the participant waits for feedback on their essay they are required to read and evaluate their “partner’s” essay in the next 5 min. “Partner” essays consist of two standardized essays reflecting either abortion stance and participants are matched to a partner essay that opposes their abortion perspectives. Following the evaluation of their partner’s essay, participants are asked to read their “partner’s” evaluation of their essay for 5 min.

### 2.4. Procedure

All procedures in the present study were reviewed and approved by the university’s Institutional Review Board. All enrolled students age 18 and above were eligible for participation.

Session 1. Following the informed consent process, the remainder of the first session was devoted to completing a battery of self-report assessments including the SNAP-2. After Session 1, participants were matched into pairs on the basis of similar personality features indicated in the SNAP-2. One member of each pair was randomly assigned into either the PASAT-C or the essay writing behavioral analogue and the other participant was matched to the remaining task.

Session 2. One week after the first session, participants were asked to return for a second session. In Session 2, participants were first instructed to complete a packet of trait-based coping and emotion regulation measures. Following these measures, participants were asked to respond to a packet of state-based measures including the PANAS, S-ERQ, and S-DERS. Next, the mood induction task (either the PASAT-C or essay) was completed. Immediately after the mood induction, participants were asked to respond to a new packet of the same state-based measures completed pre induction. Participants were then debriefed as to the purposes of the present study. To compensate participants for time spent on this study, extra credit was awarded.

## 3. Results

### 3.1. Preliminary Analyses

In the current sample, 36% of participants met criteria for a PD. Specifically, eight individuals met criteria for Antisocial PD, 14 for Avoidant PD, 17 for Borderline PD, seven for Dependent PD, seven for Histrionic PD, nine for Narcissistic PD, 19 for Obsessive Compulsive PD, four for Paranoid PD, four for Schizoid PD, and three for Schizotypal PD. Of the participants meeting criteria for a PD, 67% met criteria for only one PD (*n* = 42), 22% met criteria for two PDs (*n* = 14), 10% met criteria for three PDs (*n* = 6), and 1% met criteria for four PDs (*n* = 1). PD status was investigated as an aggregated variable (PD *vs.* no PD) rather than assessing the effects of individual PDs. Emotion dysregulation plays a role in the development and maintenance of PDs across categories of personality psychopathology. The role of emotion dysregulation in the maintenance of PD symptoms, reliance on a relatively high functioning college student population, and assessment of PD status with a self-report measure supported the investigation of PDs in general rather than emphasizing diagnostic specificity. This is of particular importance as the validity of individual PD diagnoses has been called into question due to high rates of comorbidity between PDs, substantial overlap with mood and anxiety disorders, low prevalence rates of some PDs, and wide symptom variability within PD diagnoses [37,38,39]. Thus, there is utility in investigating emotion dysregulation as it is captured by the PD category in general rather than emphasizing specific PD labels, which are not necessarily valid.

A series of ANOVA and Pearson chi-square analyses were performed to determine the differences between groups in demographic variables like age, sex, and race. Table 1 indicates no significant differences among these demographic variables and condition or among demographic variables and PD status. To confirm an even distribution of PDs across conditions, a chi-square analysis was run. No significant differences between conditions were found.

### 3.2. Experimental Manipulation Check

A repeated measures 2 (pre-induction *vs.* post-induction) × 2 (PASAT-C *vs.* essay) × 2 (PD *vs.* no PD) MANOVA was run to test the assumption that the mood induction conditions evoked negative mood which we defined as upset and afraid feelings on the PANAS. Due to positively skewed data, the upset and afraid PANAS subscales were transformed using a log10 transformation [40]. As the results were the same between the transformed and non-transformed MANOVAs, the data reported in the present study reflect the non-transformed MANOVA to provide more meaningful comparisons to the literature. The MANOVA demonstrated a significant main effect for time [Pillais’ Trace = 0.188, *F*(2, 170) = 19.61, *p* < 0.001, *partial* η^2^ = 0.19], as participants reported a statistically significant increase in feeling upset (*p* < 0.001), but not afraid *(p* > 0.05) following both mood induction conditions (see Table 2). Specifically, 19% of the variation in the dependent variable (upset feelings on PANAS) can be accounted for by time (from pre to post mood induction), indicating that the mood inductions did in fact evoke upset mood from time 1 to time 2. A follow-up repeated measures ANOVA indicated a moderate effect of mood induction on upset feelings (cohen’s *d* = 0.45).

Within this MANOVA, a significant interaction effect was found for time by condition [Pillais’ Trace = 0.083, *F*(2, 170) = 7.74, *p* < 0.001, *partial* η^2^ = 0.083] where upset and afraid were significantly impacted by mood induction condition (*p* < 0.001). As demonstrated in Table 2, follow-up repeated measures ANOVAs were run on each mood induction condition (PASAT-C and essay-writing task as independent variable) separately and revealed a significant increase in feeling upset (dependent variable) after both tasks (*p* < 0.001). The PASAT-C evoked a greater degree of upset feelings than the essay-writing task as follow-up repeated measures ANOVAs revealed a strong effect of the PASAT-C on feelings of upset (cohen’s *d* = −0.64) and a moderate effect of the essay-writing task on upset mood (cohen’s *d* = −0.27). Additionally, the PASAT-C was the only mood induction task to evoke feeling afraid (dependent variable) (*p* < 0.001) (cohen’s *d* = −0.22). Because the upset and afraid subscales are not as commonly employed as the combined PANAS negative affect scale, to test for experimental manipulation a follow-up repeated measures ANOVA was run using the combined scale and was found to be significant (*p* < 0.05). A follow-up repeated measures ANOVA found a small effect of both mood inductions on the combined negative affect scale of the PANAS (cohen’s *d* = −0.18).

**Table 2:** Repeated measures MANOVA and follow-up ANOVAs examining the relationship between time, mood induction condition, personality disorder status, and negative affect (upset, afraid, and combined upset and afraid scales).

	*F* (2, 170)	*p*	*Partial* η^2^	Mean pre (SE), Mean Post (SE)	Cohen’s *d*
Time	19.61	0.00	0.19		
PANAS_Afraid	0.04	0.85	0.00	10.06 (0.36), 10.08 (0.35)	−0.05
PANAS_Upset	30.78	0.00	0.15	7.49 (0.26), 9.09 (0.31)	−0.45
PANAS_NA	7.69	0.00	0.043	17.24 (0.59), 18.50 (0.59)	−0.19
PANAS_PA	5.77	0.02	0.033	27.51 (0.72), 26.30 (0.76)	0.11
Time X Condition	7.74	0.00	0.08		
PANAS_Afraid	11.28	0.00	0.06	PASAT-C 10.25 (0.51), 11.28 (0.50)	−0.22
				Essay 9.87 (0.52), 8.89 (0.51)	0.18
PANAS_Upset	10.03	0.00	0.06	PASAT-C 7.50 (0.38), 9.86 (0.43)	−0.64
				Essay 7.48 (0.36), 8.32 (0.44)	−0.27
PANAS_NA	11.52	0.00	0.06	PASAT-C 17.76 (0.80), 20.23 (0.78)	−0.35
				Essay 17.35 (0.80), 17.10 (0.80)	−0.10
PANAS_PA	5.67	5.67	0.12	PASAT-C 27.09 (1.00), 24.79 (1.06)	0.25
				Essay 27.93 (1.02), 27.82 (1.08)	0.02
Time X PD	0.59	0.56	0.01		
Time X Condition X PD	0.89	0.41	0.01		

### 3.3. Primary Analyses: Effects of Mood Induction Condition and PD on State Emotion Regulation

Prior to conducting a MANCOVA, all state-based emotion regulation variables (S-DERS and S-ERQ) were transformed using a log10 due to positively skewed data. The results from the transformed MANCOVA were compared to the results of a non-transformed MANCOVA. The non-transformed results were reported, as no significant differences between MANCOVAs were observed. All state based emotion regulation variables were included in a series of Pearson correlations to test the assumption that the dependent variables would be moderately correlated and appropriate for inclusion in a MANCOVA [40]. Because the S-ERQ reappraisal and suppression subscales showed weak correlations with the S-DERS subscales, the S-ERQ was not included in this analysis, but instead the subscales were analyzed with separate ANCOVAs as follow-up analyses (see Table 3 and Table 4). To control for variability in state emotion regulation before the mood induction procedure, pre-mood induction state emotion regulation subscale scores from the S-DERS were entered as covariates in the MANCOVA [40].

**Table 3:** MANCOVA and follow-up ANCOVAs examining the interaction between mood induction, personality disorder, and S-DERS with pre mood induction subscale scores of non-acceptance, goals, strategies, impulse, awareness, and clarity as covariates.

	*F* (6, 159)	*p*	*Partial* η^2^	Mean (SE) PASAT-C No PD	Mean (SE) PASAT-C PD	Mean (SE) Essay No PD	Mean (SE) Essay PD
Condition	2.80	0.01	0.10				
Personality Disorder (PD)	2.10	0.06	0.07				
Condition X PD	2.98	0.01	0.10				
Non-acceptance	4.17	0.04	0.02	9.65 (0.58)	9.37 (0.74)	10.05 (0.55)	7.10 (0.79)
Goals	0.03	0.86	0.00	10.46 (0.37)	10.77 (0.47)	10.50 (0.35)	10.65 (0.50)
Strategies	7.61	0.00	0.04	13.05 (0.45)	14.34 (0.56)	12.92 (0.42)	11.45 (0.60)
Impulse	13.59	0.00	0.08	7.93 (0.29)	9.49 (0.37)	8.15 (0.27)	7.29 (0.39)
Awareness	0.13	0.72	0.00	16.87 (0.57)	16.87 (0.57)	16.87 (0.57)	16.30 (0.76)
Clarity	7.43	0.01	0.04	8.88 (0.36)	10.00 (0.45)	8.90 (0.33)	7.85 (0.48)

**Table 4:** ANCOVAs examining the interaction between mood induction, personality disorder, and positive affect, suppression, or reappraisal with pre mood induction positive affect, suppression, and reappraisal scores as covariates.

	*F*	*p*	*Partial* η^2^	Mean (SE) PASAT-C No PD	Mean (SE) PASAT-C PD	Mean (SE) Essay No PD	Mean (SE) Essay PD
PANAS_PA	(1, 170)						
Condition	5.76	0.02	0.03				
Personality Disorder (PD)		0.59	0.00				
Condition X PD		0.03	0.03	25.82 (0.87)	24.22 (1.06)	26.03 (0.79)	28.69 (1.14)
ERQ_Suppression							
Condition		0.23	0.01				
Personality Disorder (PD)	2.52	0.12	0.02				
Condition X PD	0.06	0.81	0.00	12.99	13.75	13.52	14.56
ERQ_Reappraisal							
	0.04	0.85	0.00				
Condition							
	0.14	0.71	0.00				
Personality Disorder (PD)							
	1.58	0.21	0.01	18.35	17.75	17.37	18.47
Condition X PD				(0.60)	(0.73)	(0.55)	(0.80)

To test the hypothesis that participants meeting criteria for a PD would respond to the mood induction tasks with elevated state emotion dysregulation specific to each mood induction, a 2 (PASAT-C *vs.* essay) × 2 (PD *vs.* no PD) MANCOVA (with pre-mood induction S-DERS subscales of non-acceptance, goals, impulse, awareness, strategies, and clarity as covariates) was performed with post mood induction S-DERS subscales (non-acceptance, goals, impulse, awareness, strategies, and clarity) as the dependent variable.

A statistically significant main effect of mood induction condition was found Pillais’ Trace = 0.096, *F*(6, 160) = 2.80, *p* < 0.05, *partial* η^2^ = 0.10, suggesting that the PASAT-C evoked more dysregulation in the areas of difficulties with emotional clarity, impulse control difficulties, and strategies across participants (*p* < 0.05). A main effect for personality disorder was not demonstrated (see Table 3), suggesting that personality disorder status alone did not account for elevated state emotion dysregulation.

**Figure 1:**
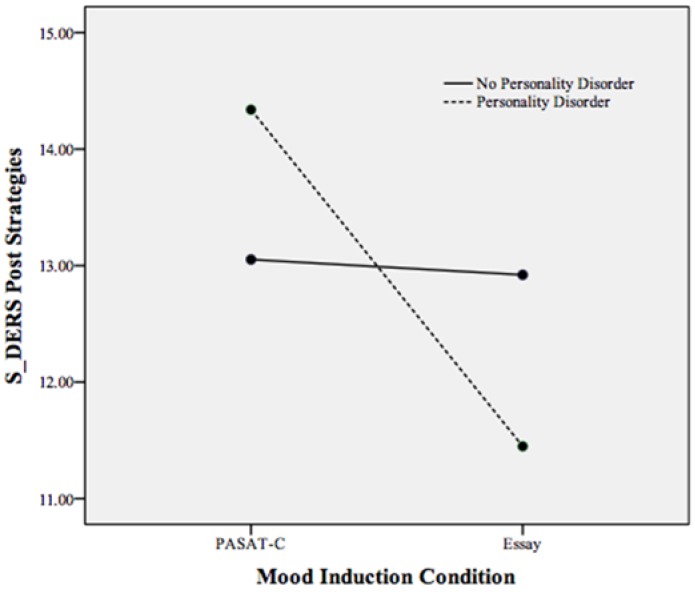
Plot of the statistically significant interaction between mood induction condition and personality disorder on the S-DERS subscale of state difficulties in emotion regulation strategies following both mood inductions. Higher scores indicate more difficulties in emotion regulation strategies.

**Figure 2:**
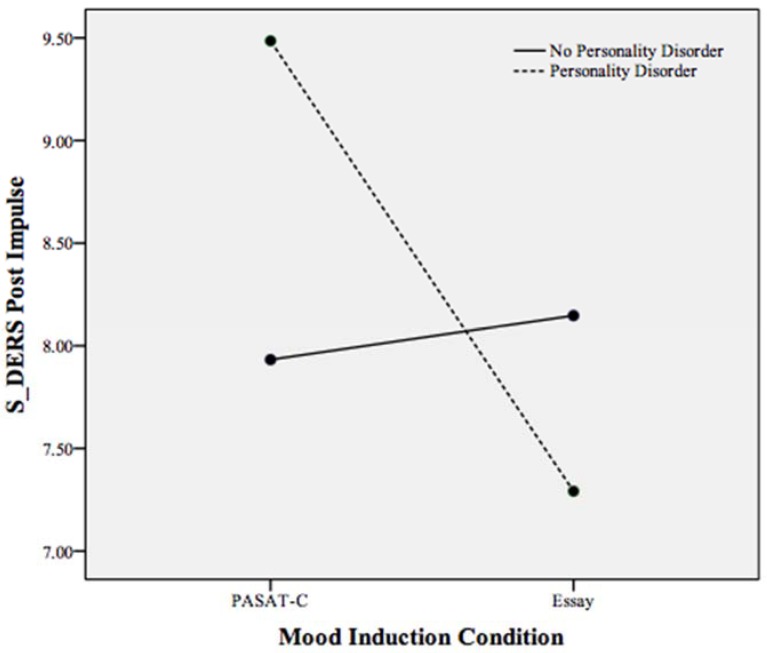
Plot of the statistically significant interaction between mood induction condition and personality disorder on the S-DERS subscale of state impulse control difficulties following both mood inductions. Higher scores indicate more impulse control difficulties.

**Figure 3:**
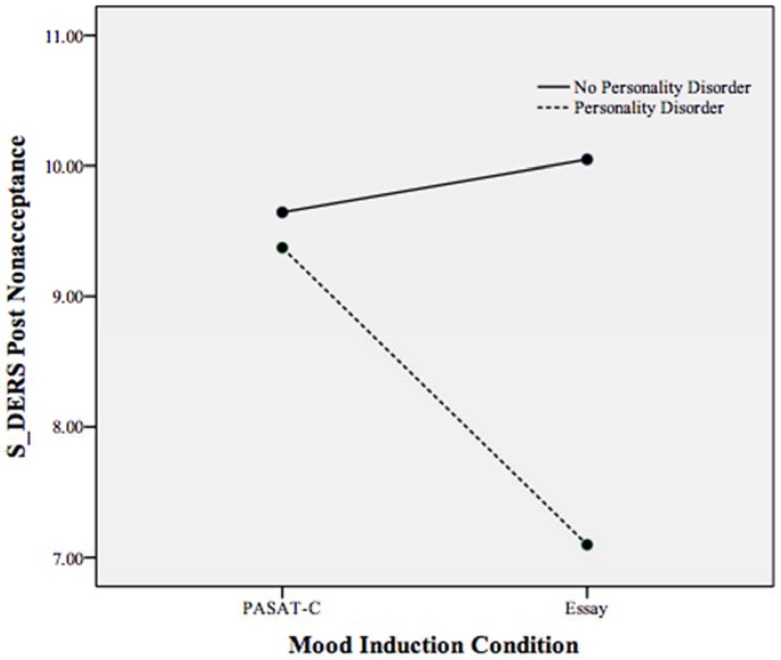
Plot of the statistically significant interaction between mood induction condition and personality disorder on the S-DERS subscale of state difficulties in non-acceptance of emotions following both mood inductions. Higher scores indicate more non-acceptance of emotions.

**Figure 4:**
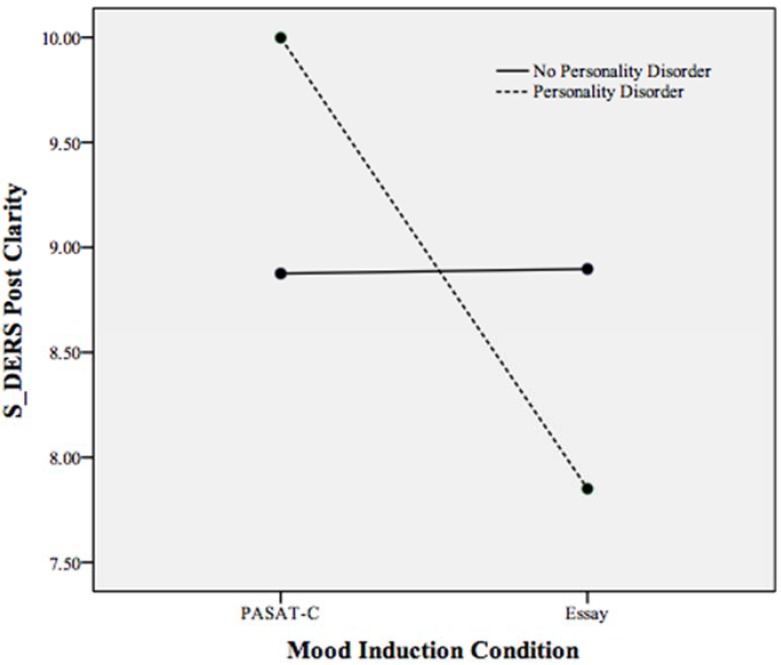
Plot of the statistically significant interaction between mood induction condition and personality disorder on the S-DERS subscale of state difficulties in emotional clarity following both mood inductions. Higher scores indicate more difficulties in emotional clarity.

A statistically significant interaction effect was found between mood induction condition and personality disorder status on state emotion regulation, Pillais’ Trace = 0.101, *F*(6, 159) = 2.98, *p* < 0.05, *partial* η^2^ = 0.101 (controlling for pre induction non-acceptance, goals, impulse, awareness, strategies, and clarity). As displayed in Table 3, non-acceptance, clarity, strategies, and impulse control demonstrated a significant interaction effect with PD status on state emotion regulation. To examine this interaction effect, follow-up ANCOVAs were run with mood induction condition and PD status as independent variables and S-DERS subscales as the dependent variable (*i.e.*, the subscales of strategies, impulse control difficulties, clarity, and non-acceptance each as a dependent variable in separate ANCOVAs). These ANCOVAs revealed that participants meeting criteria for a PD reported more difficulty accessing emotion regulation strategies (*p* < 0.001), more impulse control difficulties (*p* < 0.001), and more difficulties with emotional clarity (*p* < 0.05) compared to participants with a PD in the essay task and participants without a PD across conditions (see Figure 1, Figure 2 and Figure 3). Individuals meeting criteria for a PD in the essay-writing condition, however, reported significantly less difficulty with emotional clarity (*p* < 0.05) and non-acceptance of emotions (*p* < 0.05) following the mood induction than participants without a PD and PASAT-C participants (see Figure 3 and Figure 4).

### 3.4. Follow-Up Analyses

**Figure 5:**
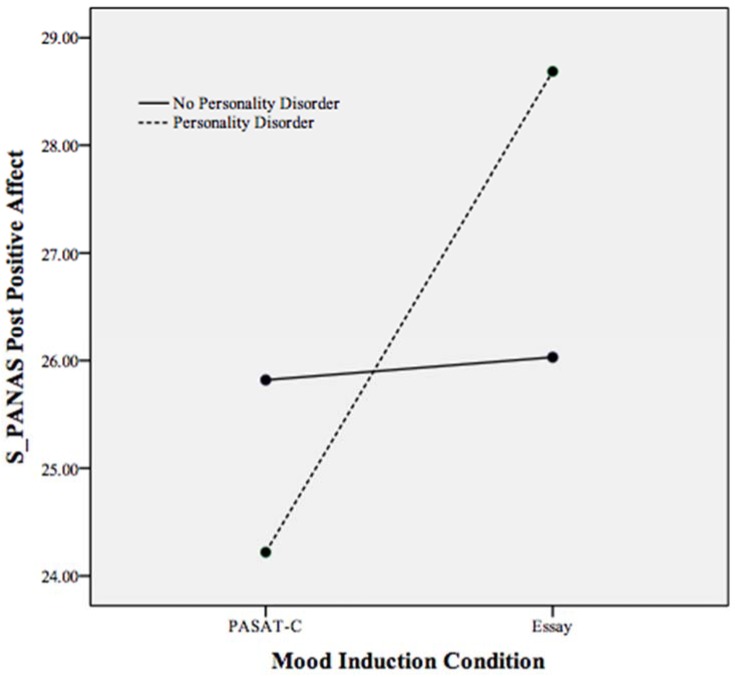
Plot of the statistically significant interaction between mood induction condition and personality disorder on the PANAS positive affect scale following both mood inductions. Higher scores indicate greater positive affect.

To understand more about individuals with a PD reporting significantly less emotion dysregulation in the essay-writing task (specifically in the areas of emotional clarity and non-acceptance of emotions) than individuals without a PD in this task, a follow-up ANCOVA for positive affect (PA) was performed. PA was assessed at follow-up due to the unexpected nature of our primary results and research suggesting that elevation of PA can be associated with affective suppression as a regulatory strategy [41]. In this ANCOVA, pre mood induction PA served as a covariate. A statistically significant main effect (*p* < 0.05) of mood induction condition was found, as PA was significantly higher in the essay-writing task across participants. However, a significant interaction effect (*p* < 0.05) was also found, demonstrating the relationship between mood induction condition, PD, and PA as individuals with a PD in the essay-writing task reported significantly higher PA following the essay-writing task than all other participants (see Figure 5). While the essay-writing task was associated with increased PA among individuals with a PD, this task did not evoke elevated PA among non-PD participants. Most participants remained consistent in PA from pre to post essay-writing task, indicating that PA was not induced across participants in response to the essay-writing task (Table 2).

**Figure 6:**
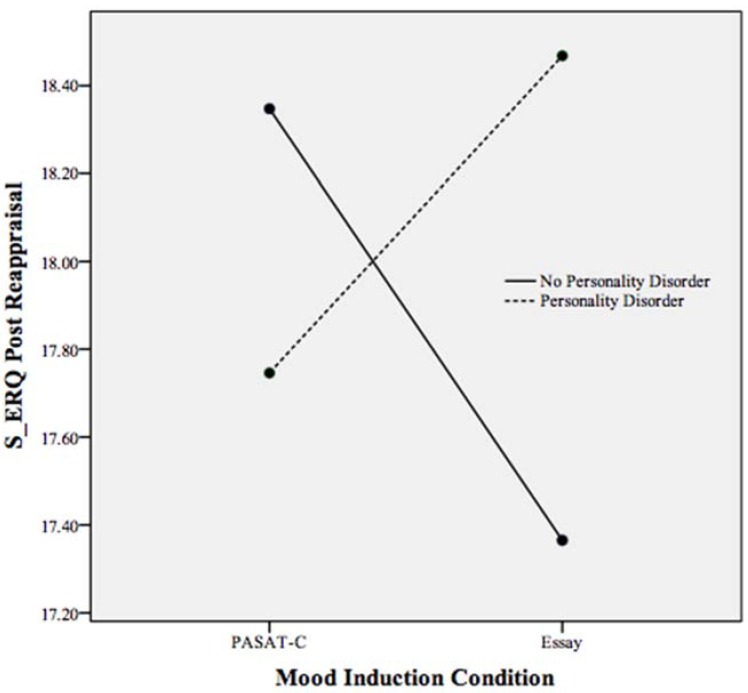
Plot of the non-statistically significant trends in the relationship between mood induction condition, personality disorder, and the reappraisal scale of the S-ERQ following both mood inductions. Higher scores indicate more use of reappraisal.

**Figure 7:**
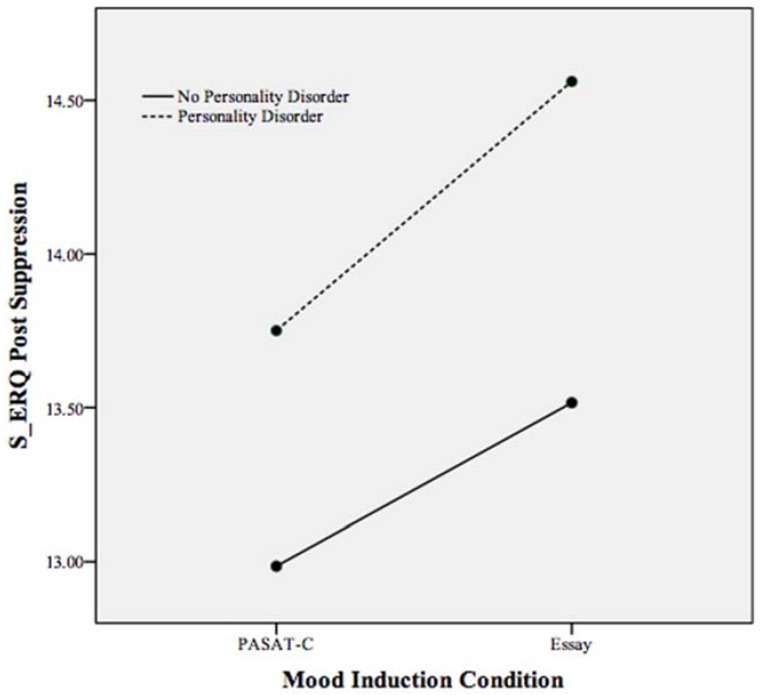
Plot of the non-statistically significant trends in the relationship between mood induction condition, personality disorder, and suppression scale of the S-ERQ following both mood inductions. Higher scores indicate more use of suppression.

As the S-ERQ subscales were not appropriate for inclusion in the MANCOVA with the S-DERS subscales, separate ANCOVAs were run to assess the effects of personality disorder and mood induction condition on the S-ERQ subscales. Elevated PA has been associated with the use of emotional suppression as a regulatory strategy [41]. To understand more about the function of heightened PA following the essay-writing task among individuals with PDs, follow-up analyses using the S-ERQ were conducted as the S-ERQ specifically measures emotional suppression. Reappraisal, the other scale of the S-ERQ, was included as a dependent variable in a separate follow-up ANCOVA with mood induction condition and PD status as independent variables. Because reappraisal is associated with changing the way a situation is perceived, the use of more reappraisal among individuals with a PD in the essay-writing task could have accounted for heightened PA. Pre S-ERQ subscale scores served as covariates. As Table 4 indicates, no statistically significant effects for condition, PD, or condition by PD were found on the suppression or reappraisal subscales. While not statistically significant, Figure 6 demonstrates a trend in the interaction between mood induction condition and PD on reappraisal (see Table 4). This trend suggests that individuals with a PD reported higher levels of emotional reappraisal following the essay-writing task when compared to all other participants. In examining the relationship between mood induction condition and suppression, a non-statistically significant trend is visible, suggesting the use of more suppression among PD participants in both conditions. Additionally, following the essay-writing task, all participants reported heightened suppression, however, this trend was not statistically significant (see Figure 7).

## 4. Discussion

Consistent with our hypotheses, individuals meeting criteria for a PD reported more state-based impulse control difficulties and lack of access to emotion regulation strategies (compared to individuals without a PD) following the PASAT-C than compared to the essay writing task. These findings not only provide further evidence that emotion dysregulation is implicated in PDs, but support elevated state emotion dysregulation as a feature of personality psychopathology. Impulse control difficulties, emotion regulation strategies, and emotional clarity were specifically heightened among individuals with a PD in response to the PASAT-C. Consistent with these findings, previous studies have demonstrated that participants with BPD terminate the PASAT-C earlier than healthy controls, thus displaying a lower level of willingness to tolerate distress [16]. Perhaps, difficulties with emotion regulation strategies and lack of impulse control and emotional clarity increase an individual’s vulnerability to discontinuing goal directed behavior when distressed. Based on these findings, tasks like the PASAT-C may present specific value in teaching individuals with PDs to observe and describe emotions, apply new emotion regulation strategies, and to practice non-reactivity to impulses in an immediately disruptive context.

While the essay-writing task evoked negative mood, it was not as powerful in inducing upset feelings as the PASAT-C. It was expected that individuals meeting criteria for a PD would demonstrate heightened emotion regulation difficulties in response to this mood induction. Findings from this study, however, indicate the opposite. Compared to individuals without a PD, in the essay task participants meeting criteria for a PD demonstrated significantly less difficulty with non-acceptance and clarity immediately following the mood induction. This was surprising as the very label of a PD is often equivocated with interpersonal dysfunction. When the PD group’s performance on the essay writing task is interpreted in combination with significantly greater positive affect and a trend towards increased suppression, one possible explanation is that these participants engage in more suppression than non-PD participants. In keeping with these results, Chapman, Rosenthal, and Leung [41] found that individuals high in BPD symptoms reported greater positive emotions on days they were instructed to suppress emotions. These findings are consistent with our results and may point to the reinforcing nature of emotional suppression. Lending further support to this hypothesis is research indicating that suppression is associated with decreased negative emotion in response to emotionally charged film clips. Investigators found that while suppression facilitated an immediate decrease in negative emotion, elevated brain activity in the amygdala and insula was sustained [42]. In the short term, suppression facilitated a decrease in distress, which might be demonstrated in the present study via an increase in positive emotions.

The persistent physiological arousal found in Goldin *et al.*’s [42] study points to the possibility that while suppression might facilitate decreased negative emotion in the short term, continuing distress may result in the long term. Had the present study employed a longitudinal sampling approach, emotion dysregulation could have been measured after the effects of suppression dissipated. Associated with suppression, alexithymia may be another component of emotion dysregulation that affected the results in the essay-writing task [43]. It could be that individuals meeting criteria for a PD do not have the requisite skills to identify and describe their emotion regulation strategies in the essay-writing task. Elevated (though non-significant) reappraisal of emotions and decreased reported difficulties with emotion regulation support this hypothesis. Additionally, suppression could facilitate emotional avoidance so much so that inappropriate emotional states are sought and identified (e.g., positive affect) to reduce dysregulation.

The nature of the essay-writing mood induction task might also uniquely affect individuals with a PD. Individuals meeting criteria for a PD, for instance, could be more resilient to certain types of interpersonal rejection due to frequent environmental invalidation. When the findings from the present study are interpreted based on Gross & John’s [32] model of emotion regulation and in keeping with the trend towards increased reappraisal following the essay-writing task, it might be that individuals with PDs are more readily able to use reappraisal to regulate their emotions in some interpersonal contexts (Figure 6). Rehearsal of emotion regulation strategies in similar invalidating environments may lead to the adaptive use of regulatory skills in situations such as this one.

Some research indicates that individuals with PDs do not respond differently to tasks involving social rejection than individuals without PDs. In a study assessing the effects of ostracism through a social rejection oriented computer game, individuals with BPD did not report more emotional distress following the task when compared to controls without BPD [22]. As evidenced in the BPD literature, it may be that interpersonal hypersensitivity represents a phenotype of personality psychopathology rather than a necessary trait [44]. Additionally, interpersonal sensitivity may be dependent on the context of social rejection and an individual’s social learning history. Rejection from a stranger may be less salient to individuals with a PD than rejection from a known person.

To our knowledge, this is the only study thus far to assess state emotion dysregulation in response to two contextually unique mood induction tasks. The findings from the present study are important as they indicate that different tasks evoke different emotion regulation strategies among individuals meeting criteria for a PD. The present study’s results may encourage novel treatment approaches. Based on emotion regulation deficits observed in different mood induction tasks, specific emotion regulation skills could be emphasized in therapy. Another contribution of this study is in the assessment of emotional responding in PDs. Personality psychopathology was assessed as an aggregated variable in the present study instead of focusing on differences between PDs. Studies like this one may lead to a better understanding of the general regulatory dimensions underlying personality psychopathology which could enhance the assessment and transdiagnostic treatment of PDs.

## 5. Limitations

While the present study includes a number of strengths and makes important contributions to the emotion regulation and personality disorder literature, its limitations should also be considered. One of the most notable limitations was the reliance on a relatively homogenous undergraduate sample. Based on the volume of participants included in the present study, we implemented a self-report assessment of personality psychopathology, which may have contributed to an inflation of PD diagnoses in our sample. Because we were focused on personality psychopathology, we did not employ assessments of mood and anxiety disorders, diagnoses which are also associated with emotion dysregulation [45,46,47]. Future studies should replicate this methodology in more diverse clinical contexts and account for different forms of psychopathology. Another weakness of the current study is that the behavioral analogues evoked different negative emotions, as “afraid” was only induced by the PASAT-C. The state emotion dysregulation participants reported following the PASAT-C may have been less a function of the context of the task and more heavily influenced by the degree of emotion induced by this task (*i.e.*, heightened impulsivity following the PASAT-C may be due to the amount of negative emotion induced rather than the type of task). Magnitude of negative mood evoked may have also played a role in the essay-writing task, as this mood induction was not as robust as the PASAT-C. The upset feelings induced by the essay-writing task may not have been strong enough to evoke emotion dysregulation to a degree that could be identified among individuals with PDs who are persistently dysregulated. In the future, when comparing two mood inductions, researchers should attempt to make the two tasks as similar as possible to avoid potential confounding variables influencing state emotion regulation and to increase the generalizability of findings. Additionally, more ecologically valid mood inductions might be implemented where saliency in a laboratory context is traded for tasks that more closely resemble daily life stressors. Another limiting factor is that both mood induction procedures relied on self-report measures to assess state emotion regulation. In the future, physiological measures of emotion in tandem with behavioral observations might provide even more information about state emotion dysregulation.

## 6. Future Directions

In order to assess the potential role of suppression in social rejection scenarios, future studies should measure emotion dysregulation at different time points related to mood induction tasks. If emotion dysregulation in interpersonal contexts is delayed due to suppression, vulnerability to increased dysregulation could result in the long-term. Also important to future research is the continued development of realistic interpersonal mood inductions so that the effects of social rejection on emotion dysregulation in PDs can be most accurately examined.

## 7. Conclusions

Based on the findings from the present study, different mood induction conditions evoke different emotion regulation strategies among individuals meeting criteria for PDs. As expected, following the PASAT-C, individuals meeting criteria for a PD reported significantly greater difficulty with impulsivity and access to emotion regulation strategies than all other participants. After the essay writing task, however, individuals with a PD reported significantly fewer difficulties with emotional clarity and nonacceptance of emotions than individuals without PDs. Individuals with PDs in the essay writing task also reported significantly greater positive affect following this mood induction when compared to all other participants. This study demonstrates that individuals with PDs respond to different environmental contexts with different state emotion regulation difficulties. Furthermore, these difficulties are not apparent in individuals without PDs, suggesting the role of personality psychopathology in elevated state emotion dysregulation. Given these findings, continuing this research is essential as the context in which state emotion dysregulation occurs and the type of dysregulation presented could have meaningful implications for the assessment and treatment of personality psychopathology.
